# Weed Warden: A low-cost weed detection device implemented with spectral triad sensor for agricultural applications

**DOI:** 10.1016/j.ohx.2022.e00303

**Published:** 2022-04-01

**Authors:** Liam Duncan, Brendan Miller, Colton Shaw, Ryan Graebner, Marcelo L. Moretti, Cara Walter, John Selker, Chet Udell

**Affiliations:** a Openly Published Environmental Sensing (OPEnS) Lab, OR, USA; b Department of Electrical Engineering and Computer Science, Oregon State University, OR, USA; c Columbia Basin Agricultural Research Center, Department of Crop and Soil Science, Oregon State University, OR, USA; d Department of Horticulture, Oregon State University, OR,USA; e Department of Biological and Ecological Engineering Oregon State University, OR, USA

**Keywords:** Open-source hardware, Environmental sensing, Spectral sensing, Spectroscopy

## Abstract

Controlling weeds is essential for farmers to protect resources and maximize crop yield. Between crops, weeds are typically controlled by applying herbicides or tillage to the entire field. However, these control methods are expensive and can pose environmental risks. Robotic weeding systems are a good solution to minimize environmental impact and save money on herbicides, but they are expensive (>$100,000). The Weed Warden is a low-cost (<$200) plant detection sensor that can be mounted on rovers or tractors. The Weed Warden uses an open source multispectral sensor to detect live vegetation and sends a logic signal that could trigger a weed removal system such as a sprayer or mechanical tillage when vegetation is detected. We evaluate the Normalized Difference Vegetation Index (NDVI), Enhanced Normalized Difference Vegetation Index (ENDVI), and Enhanced Vegetation Index (EVI), for producing a value that, combined with a calibrated threshold, will indicate if there is plant life under the sensor. The Weed Warden system using ENDVI is most consistent at detection, with the ability to discriminate 7.6x7.6 cm vegetation samples from bare soil at sensor heights of 30 and 41 cm from the ground. The Weed Warden is a proof-of-concept component of an alternative system to robotic weeders of fallow fields that could help reduce costs, improve environmental outcomes in agricultural settings, and advance research into fallow field management practices.

## Graphical abstract

**Figure:**
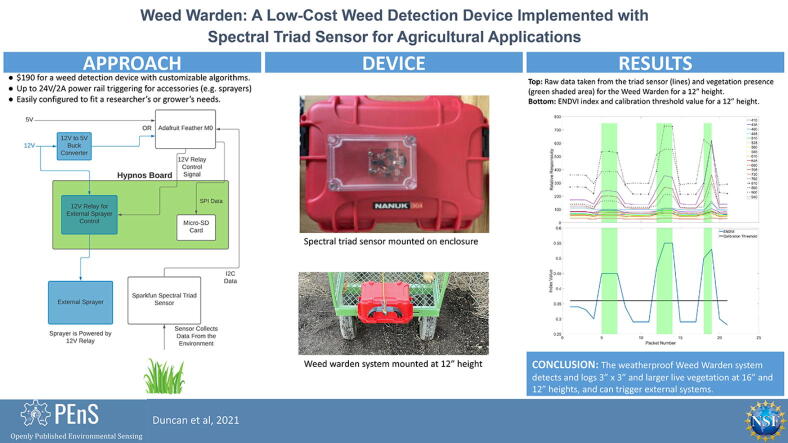

**Specifications table**

**Table:** 

Hardware name	*Weed Warden*
Subject area	Environmental, Planetary and Agricultural Sciences
Hardware type	Field measurements and sensors
Closest commercial analog	Weed-It
Open Source License	CERN Open Hardware License GNU General Public License v3.0
Cost of Hardware	$ 189.17
Source File Repository	*https://doi.org/10.5281/zenodo.4724135*

## Hardware in context

In many agricultural systems, fields must be kept free of plants during a fallow year between crops, including both weeds and volunteer plants from the previous crop. This fallow period can conserve moisture for the following crop, provide a disease break, and help control problem weeds. Maintaining plant-free fallows is most commonly achieved by applying chemical control (herbicides) or mechanical control (tillage) to the entire field, even though in many cases there may be fewer than 100 plants that need to be controlled per acre. In addition to the added cost of applying control methods to the entire field, herbicides can carry environmental and human health risks, while tillage is fuel-intensive, can degrade soil quality, and can increase soil erosion [1].

A promising route to reduce the financial, environmental, and human health consequences of weed control during the fallow period is to target control efforts to individual weeds, limiting the total area where weeds need to be controlled. This is the focus of several robotic weeding systems that are currently in development. However, commercial weeding robots such as the Naio Dino are expensive (>$100,000) and require specialized equipment [2]. Other notable commercial systems are Weed Seeker by Trimble [3] and Weed It Quadro. Like Naio Dino, cost is a major barrier that may prevent wide adoption of this technology.

In contrast, the Weed Warden, a system for sensing plants and triggering external equipment, is low cost (<$200) and modular. The Weed Warden uses a low-cost multispectral sensor, calibrated threshold, and vegetation index to detect green plants with the sensor placed up to 41 cm off the ground. The Weed Warden is designed with the intent to be attached to any equipment that the farmer already owns (e.g. tractor) or put onto a dedicated rover to remove weeds autonomously. This would allow farmers to minimize costs by adapting equipment that they already own, while still getting the cost and environmental benefits of spot spraying. Open-source portable spectrometer sensor systems like [4] and [5] have been explored for measuring a wide variety of materials both in and outside of traditional lab spaces. Some off-the-shelf hyperspectral scanners have been adapted to CNC machines for measuring plant physiology including [6], which uses the Nano line (1-pixel wide push broom style) scanner. The closest *in-situ* example of a multispectral sensor to the task of plant identification for spot application of herbicides we could find was [7], which used a lens with a narrow area on one axis, and a wide area on another to channel light into an imaging spectrograph and camera to calculate a vegetation index (normalized difference vegetation index, NDVI). Unfortunately, the exact hardware components, design files, or assembly for [7] were not detailed. Our application uses an even lower-cost multispectral sensor and an algorithm to specifically calculate spectral characteristics that indicate the presence of chlorophyll to indicate whether a living plant is present, or not. A multispectral sensor is different from a hyperspectral sensor in that the former uses a fewer number of frequency bands to represent the spectrum. Where some sensors like [8] have a 15 nm range across 450 to 750 nm for about $190 per sensor, we evaluate one that detects 18 frequency bands across 410 to 940 nm for $64. Our evaluation of this sensor indicates that these 18 bands alone are effective enough to calculate the indicators to discriminate for live vegetation detection relative to bare soil. The sensor in the Weed Warden is also different in that it is a 1-pixel “cone” representing the average spectrum within its field of view. The relationship between height of camera, field of view and accuracy of the algorithm is described in the Validation section.

## Hardware description

**Fig. 1:**
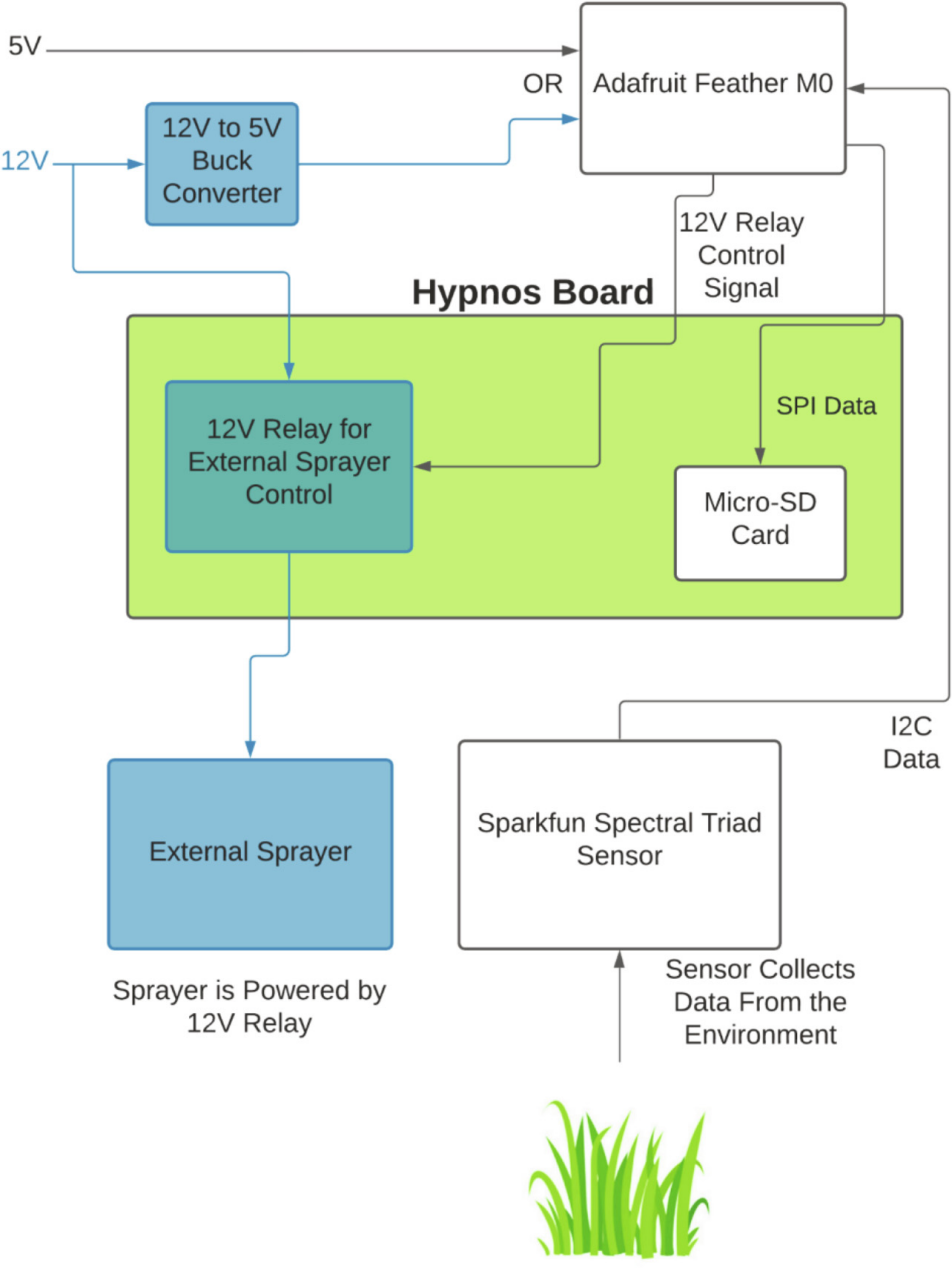
Block Diagram of the System. System can be powered by a 5 V USB, 3.7 V battery, or through a 12 V buck converter. Items in blue are associated with 12 V power.

**Fig. 2:**
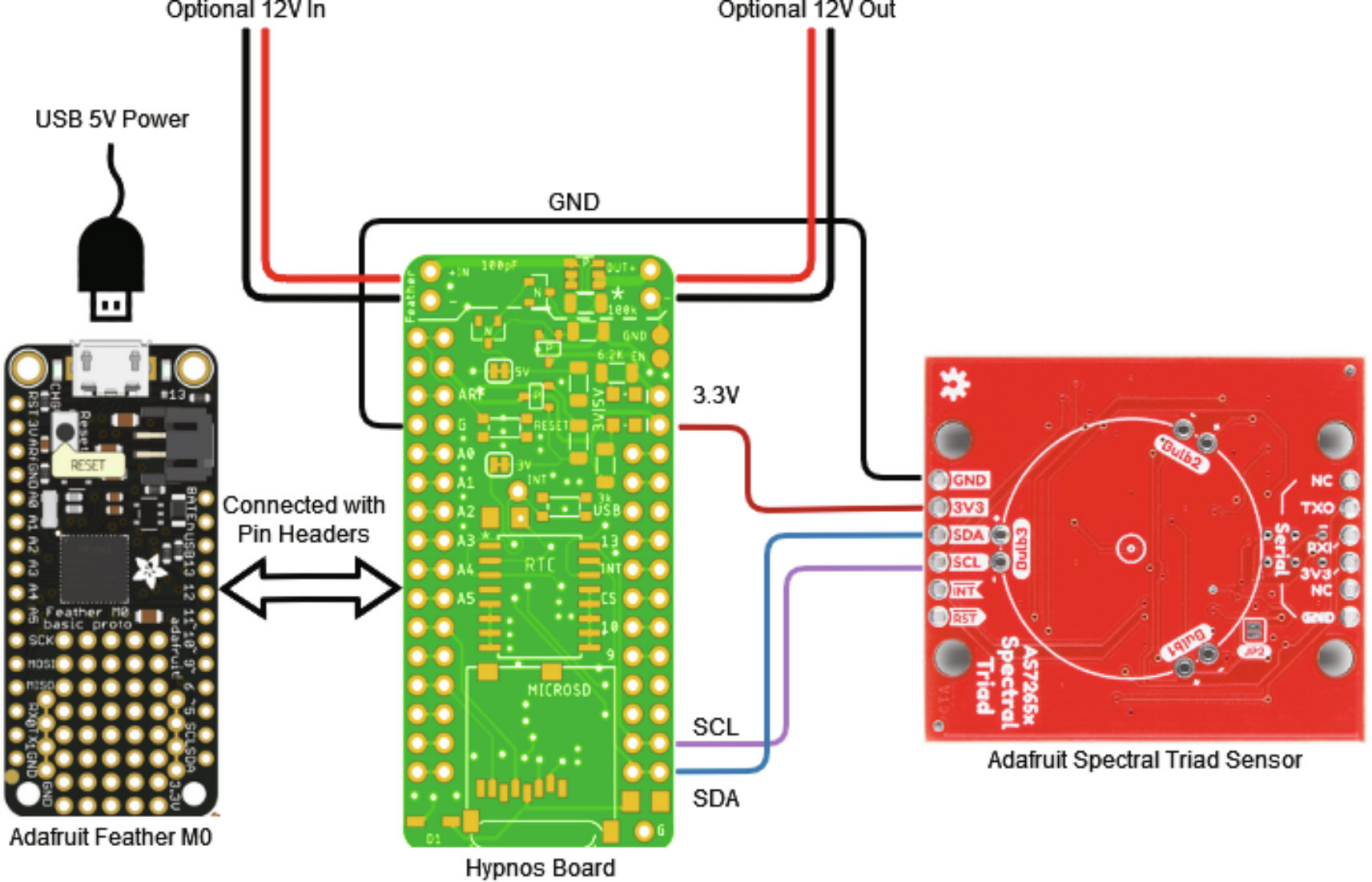
Wiring diagram for system showing connections between the Feather M0 (left), Hypnos Board (center), and Triad Sensor (right).

The Weed Warden system detects live plants, and activates external devices for targeted weed control (Fig. 1). The Weed Warden electronics consist of 2 stacked printed circuit boards (PCBs) and the AS7265x Spectral Triad Sensor [8] (Fig. 2). The main PCB assembly consists of an Adafruit Feather M0 board [9] with an OPEnS Lab Hypnos board [10] stacked on top. The Feather M0 serves as the microprocessor and the Hypnos provides power management, Real-Time Clock (RTC), and data logging.

**Fig. 3:**
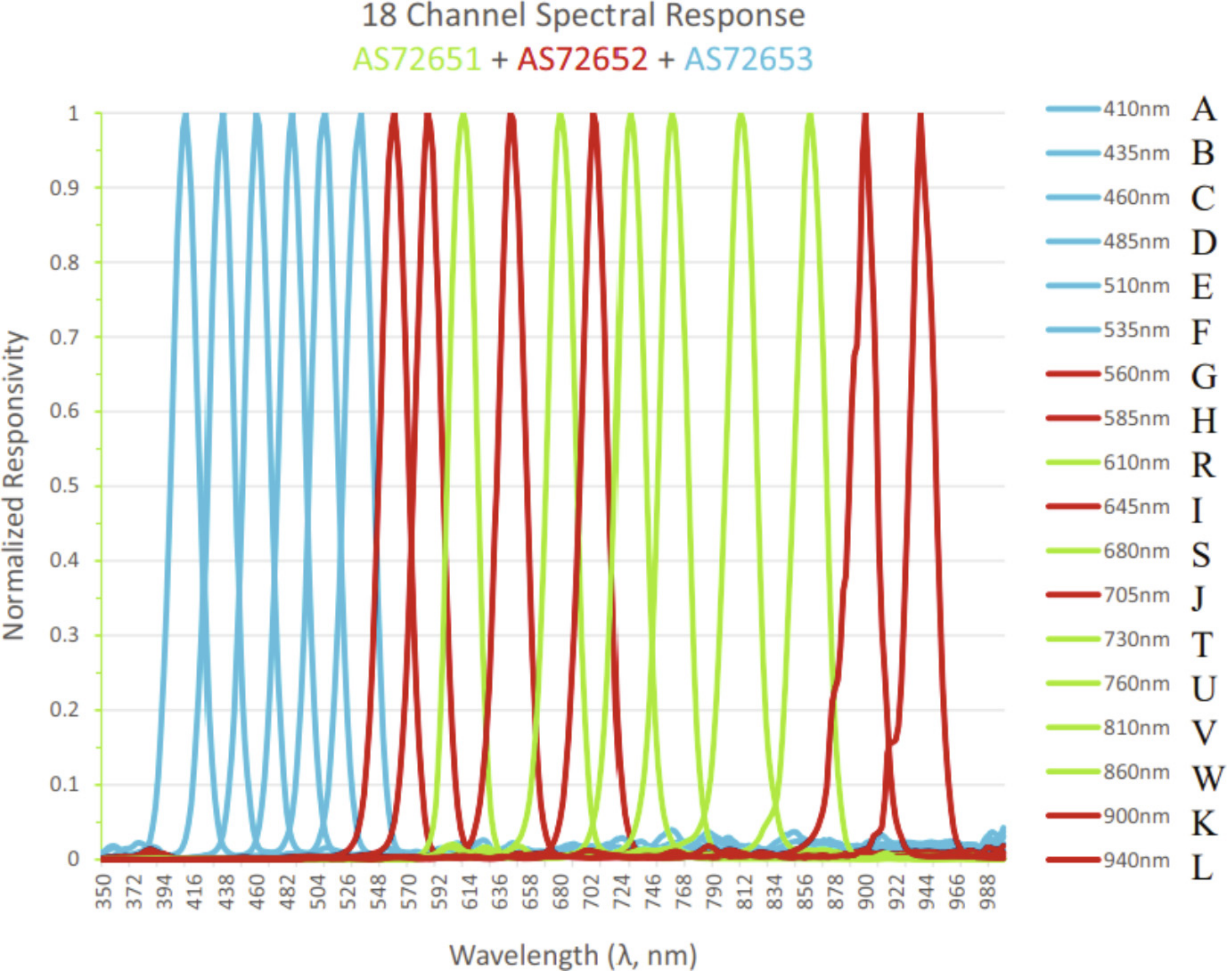
Triad sensor spectral response chart from Triad Sensor Datasheet [8]. Letters on the far right were added and correspond to variable names associated with the responsivity returned by the sensor for each wavelength in the Weed Warden code.

**Fig. 4:**
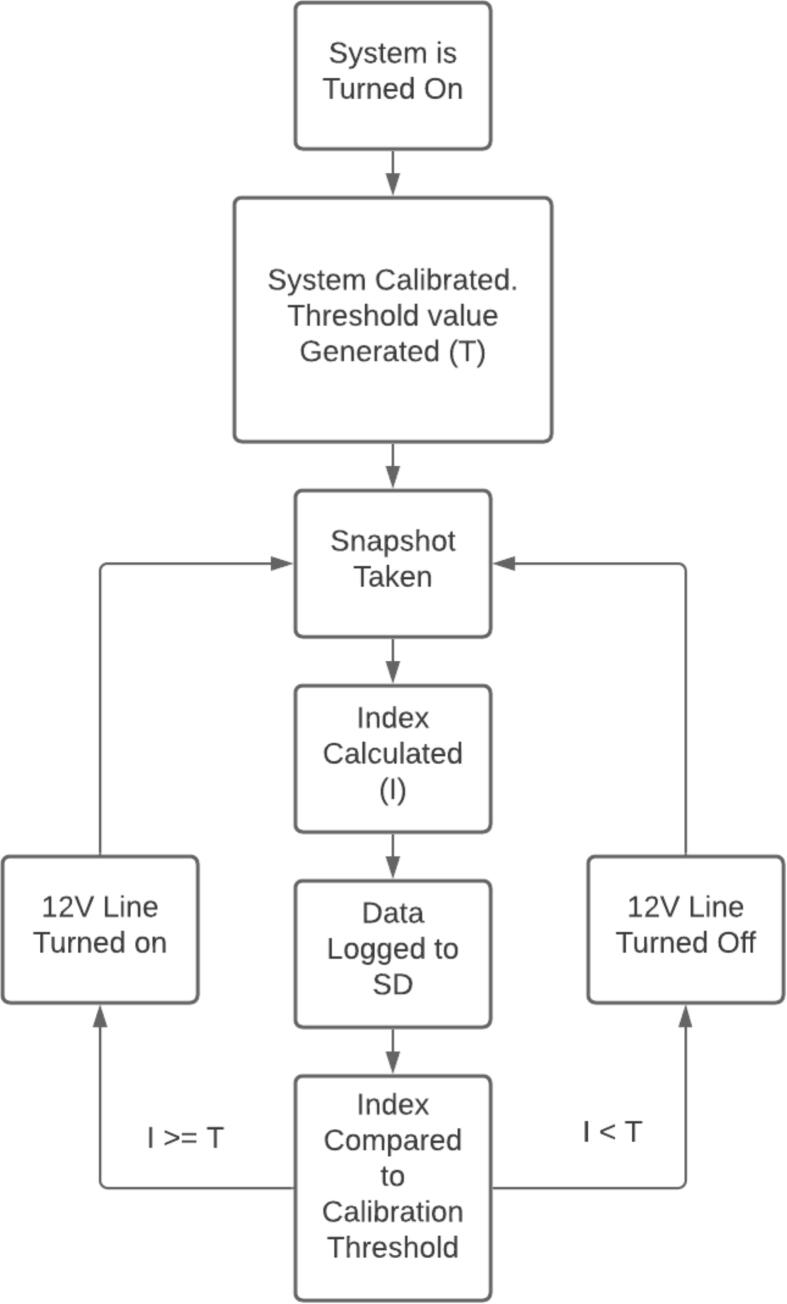
State Machine Diagram for Weed Warden Arduino code. Each cycle takes approximately 2 s.

The Weed Warden senses 18 different frequencies of light (Fig. 3), and detects plants based upon an index threshold established during pre-deployment calibration (Fig. 4). The code is written in Arduino using the Loom library developed by OPEnS Lab [11] that enables rapid prototyping of sensors and data logger hardware for environmental sensing purposes. We tested several different vegetation indices to determine the most effective indicator for the presence of a living plant including the enhanced normalized difference vegetation index (ENDVI) [12] (Eq. (1)), normalized difference vegetation index (NDVI) [13] (Eq. (2)), Enhanced Vegetation Index (EVI) [14] (Eq. (3)), and pigment specific normalised difference (PSND) [15]. The indices use varying combinations of wavelengths absorbed (visible red and blue) or reflected (near infrared (NIR) and visible green) by vegetation [12]. Based upon our results, the recommended indices for using the Weed Warden are the ENDVI or NDVI with the default set to ENDVI. Custom indices can be used to tune the Weed Warden for specific scenarios and applications.

(1) $$\mathrm{ENDVI}=\frac{(NIR+Green)-2*Blue}{(NIR+Green)+2*Blue}=\frac{R_{760}+R_{810}+R_{860}+R_{510}+R_{535}+R_{560}-2*\left(R_{435}+R_{460}+R_{485}\right)}{R_{760}+R_{810}+R_{860}+R_{510}+R_{535}+R_{560}+2*\left(R_{435}+R_{460}+R_{485}\right)}$$

(2) $$\mathrm{NDVI}=\frac{\mathrm{NIR}-Red}{\mathrm{NIR}+Red}=\frac{R_{810}-R_{680}}{R_{810}+R_{680}}$$

(3) $$\mathrm{EVI}=2.5*\frac{\left(NIR-Red\right)}{\mathrm{NIR}+6*Red-7.5*Blue+1}=2.5*\frac{\left(R_{810}-R_{680}\right)}{R_{810}+6*R_{680}-7.5*R_{435}+1}$$

The plant detection threshold value is calibrated for each deployment to account for soil and light conditions at the time of data collection. The Weed Warden system is not designed to distinguish between different types of vegetation. Because soil and light conditions will change throughout the day, periodically executing the calibration routine is advisable to update the system with the latest soil and light condition. The requirement to do this less often is possible by creating a canopy over the device to regulate ambient light, but this is beyond the scope of our sensor system.

The Weed Warden can be powered by 5 V USB, 3.7 V Adafruit Lithium Ion Battery, or through a 12 V Power Jack with a 5 V buck converter. The Weed Warden used in this article was powered by 5 V USB. The Hypnos board offers full control of 3.3 V and 5 V power rails as well as an external power supply up to 24 V/2A that can be used to toggle a sprayer or any other device.

The Weed Warden is housed in a waterproof case with a polycarbonate window for the Triad Sensor. The material of the window does not interfere with wavelengths used and is even used in many greenhouse applications [16].

The Weed Warden system provides:

● Sensing of 18 different wavelengths (Fig. 3). Ability to expand the system by adding more spectral triad sensors.
● Plant detection with multiple vegetation index analysis options including ENDVI, NDVI, EVI, PSND or custom.
● Up to 24 V/2A power rail triggering for accessories such as sprayers for weed removal, RGB cameras for vegetation documentation, or GPS receivers for vegetation location tagging.
● Micro-SD data logging (Table 1).

**Table 1:** Summary of data logged to microSD. System logs the intensity of all 18 wavelengths, three different index values, packet numbers for each data sample, and the time at which each sample was taken.

Data Type	Data Logged	Units
Light Intensity	410, 435, 460, 485, 510, 535, 560, 585, 610, 645, 680, 705, 730, 760, 810, 860, 900, 940	Relative Responsivity
Indicator Index	ENDVI, EVI, NDVI	Unitless
Packet Number	Integer Increment (approximately 2 s apart)	Integer
Timestamp	Time and date of data sample	Month/Day/Year, Hours, minutes, seconds
● Sampling frequency as short as two seconds
● Small form factor allows it to be mounted on many different devices

## Design files

### Design files Summary

**Table:** 

Design file name	File type	Open source license	Location of the file
WeedSeekerMain.ino	.ino	GNU General Public License v3.0	https://zenodo.org/record/4724135
config	.h	GNU General Public License v3.0	https://zenodo.org/record/4724135

● WeedSeekerMain.ino: The Arduino code is the code that must be uploaded to the Feather M0 to use the Weed Warden. The code contains the calibration sequence and plant detection, data logging, and Hypnos Relay control.
● config.h specifies parameters for Loom components

## Bill of materials

**Table:** 

Designator	Component	Number	Cost per unit - USD	Total cost - USD	Source of materials	Material type
Feather M0	Feather M0	1	$19.95	$19.95	Adafruit	Other
Triad Sensor	Spectral Triad Sensor	1	$64.95	$64.95	SparkFun	Other
Hypnos Board	OPEnS Lab Hypnos Board	1	$33.00	$33.00	OPEnS Lab	Other
Coin cell battery^1^	Coin Cell Battery	1	$1.95	$1.95	Spark Fun	Other
Hard Case^6^	Nanuk 904 Waterproof Hard Case with Foam Insert	1	$35.19	$35.19	Amazon	Polymer
12 V Buck Converter^2^	UBEC DC/DC Step Down Converter	1	$9.95	$9.95	Adafruit	Other
M3 Long Screws	18–8 Stainless Steel Pan Head Phillips Screws M3 × 0.5 mm Thread, 20 mm Long	1	$8.29^3^	$8.29	McMaster Carr	Metal
M3 Short Screws	18–8 Stainless Steel Hex Head Screw M3 × 0.5 mm Thread, 12 mm Long	1	$10.71	$10.71	McMaster Carr	Metal
M3 Hex Nuts	Thin Steel Hex Nut M3 × 0.5 mm Thread	1	$3.51^3^	$3.51	McMaster Carr	Metal
JST Connector	4 Pin JST XH connectors with female 300 mm wire	1	$15.99^4^	$15.99	Amazon	Other
Female Pin Headers	Header Kit for Feather 12 Pin and 16 Pin Female Header Set	2	$0.95	$0.95	Adafruit	Other
Male Pin Headers	Short Feather Male Headers − 12-pin and 16-pin Male Header Set	1	$0.50	$0.50	Adafruit	Other
Micro SD Card	SanDisk Ultra 32 GB UHS-I/Class 10 Micro SDHC Memory Card	1	$8.45	$8.45	Amazon	Other
Sensor Cover^7^	Flanged Weatherproof with PG-7 Cable Glands^5^	1	$9.95	$9.95	Adafruit	Polymer
RTV Silicone Adhesive	Clear RTV Silicone Adhesive	1	$2.99	$2.99	JB Tools	Other

^1^Optional component. This component is for the Hypnos RTC and is only necessary if using logged time.

^2^Optional component. Use this converter if a 12 V Power Source is being used to power the Weed Warden.

^3^Pack of 100.

^4^Pack of 15, only 1 female and male connector required.

^5^Only need the lid from the box.

^6^Minimum required internal dimensions for the hard case:

**Table:** 

Height	Length	Width
4 cm	6 cm	3 cm

^7^Minimum required internal dimensions for the sensor cover:

**Table:** 

Height	Length	Width
1 cm	5 cm	4 cm

## Build instructions

### Wiring overview

The Feather M0 and Hypnos Board are connected to each other via headers while the Triad sensor is connected to the Hypnos Board with a JST connector on the Triad sensor board and soldered wires on the Hypnos Board (Fig. 2).

### PCB assembly

The first step to assemble the Weed Warden is to assemble the three PCBs. Weed Warden PCB assembly involves soldering; please follow soldering safety guidelines to reduce the intrinsic hazards, hot elements and lead exposure, associated with soldering.

#### Feather M0 assembly

**Fig. 5:**
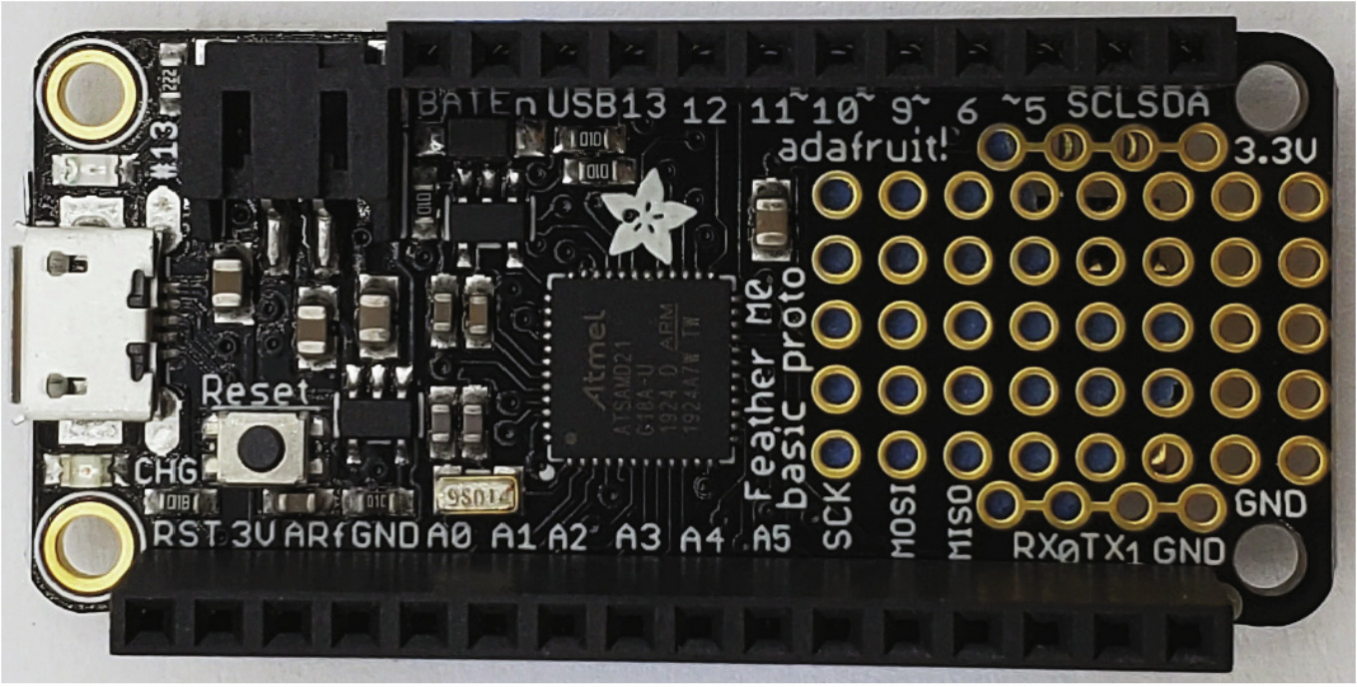
Feather M0 top side with female headers.

To assemble the Feather M0, the female pin headers must be attached to the top of the Feather M0 PCB (Fig. 5). The official Adafruit guide provides the instructions for Soldering on Female Headers [17].

#### Hypnos Board assembly

The next PCB that must be assembled is the Hypnos Board. The official Hypnos GitHub wiki contains the official build guide which should be followed when assembling the Hypnos Board [18]. The build guide covers how to place all SMD components onto the Hypnos board.

**Fig. 6:**
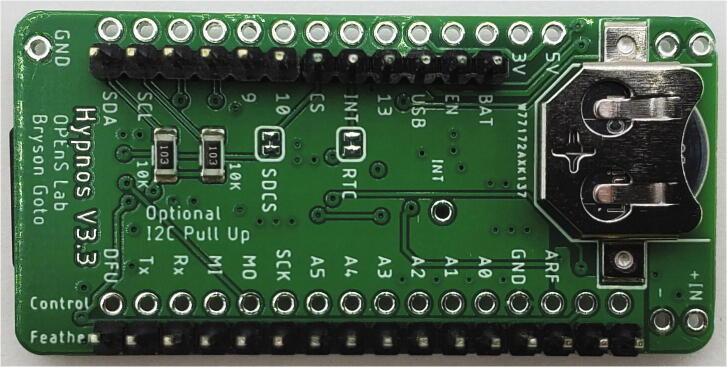
Bottom side of Hypnos Board assembled with male pin headers on Feather rails and coin cell battery inserted.

**Fig. 7:**
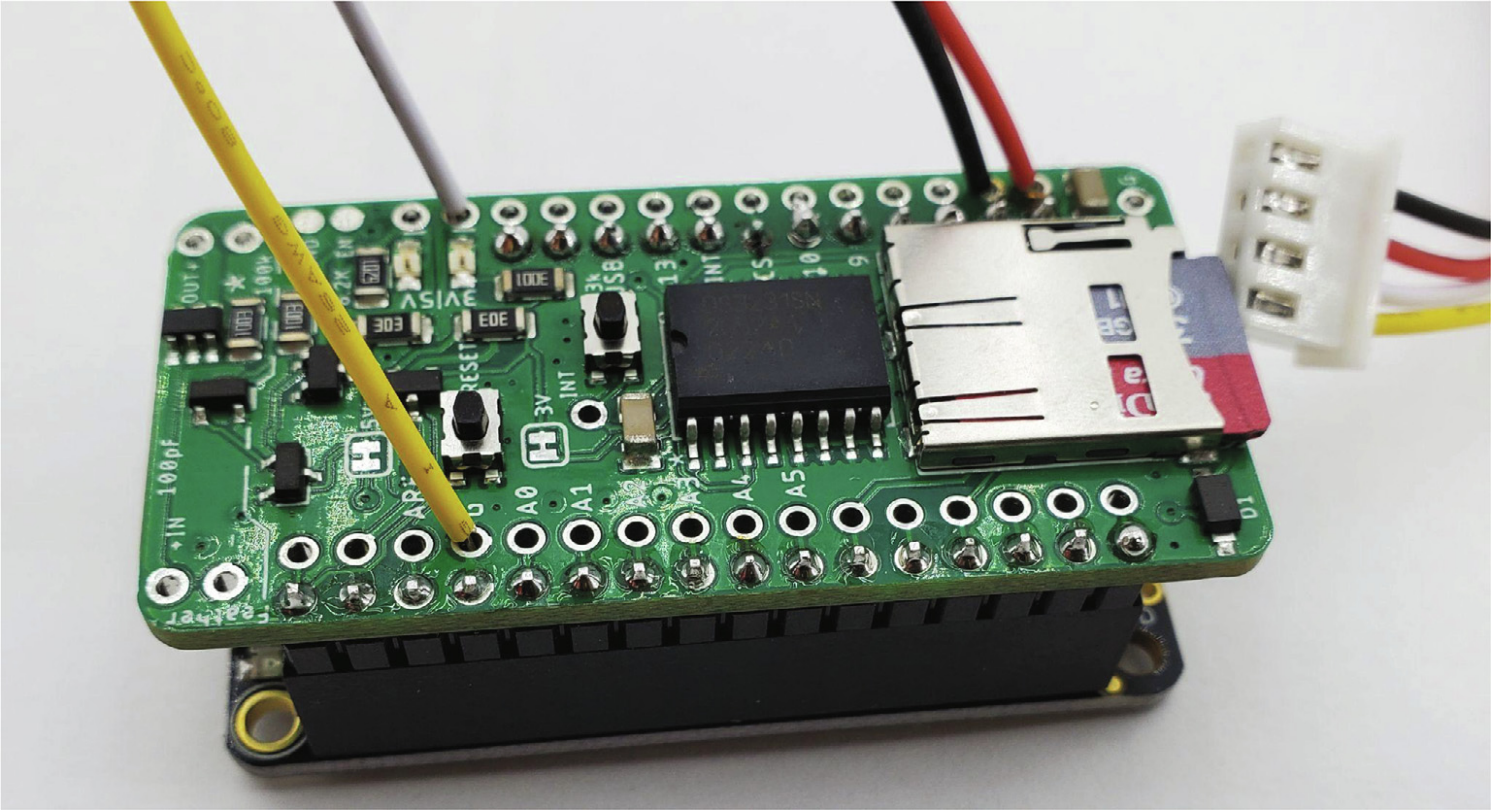
Hypnos Board stacked on top of Feather M0 with JST wires. Note that wire colors correspond with the male JST orientation on the Triad sensor shown in Fig. 8.

Next, solder male pin headers to the Feather lines (Fig. 6). Solder the JST wires to the appropriate pins on the control rail of the Hypnos Board (Table 2, Fig. 7). Insert the coin cell and microSD card into the appropriate slots (Fig. 6, 7), and stack the Hypnos on top of the Feather M0 (Fig. 7).

**Table 2:** JST wire coloring from Triad Sensor to Hypnos.

Wire color	Triad Sensor label	Hypnos label
Yellow	GND	G
White	3 V3	3 V
Red	SDA	SDA (label on back)
Black	SCL	SCL (label on back)

#### Triad sensor assembly

**Fig. 8:**
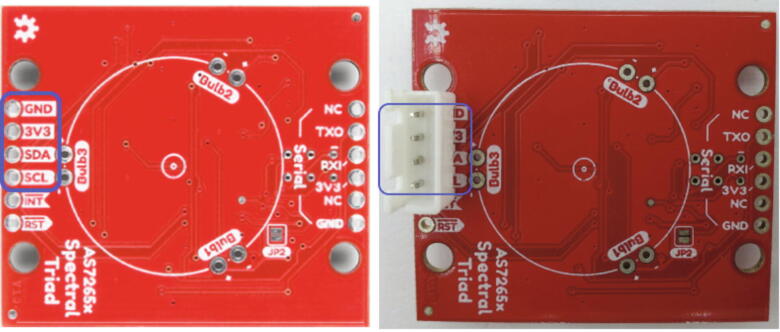
Left: Back of Triad Sensor with no components added. Pins for the JST connector are circled in blue. Right: Back of Triad Sensor with JST connector attached. JST connector pins are circled in blue.

The last PCB that must be assembled is the Triad sensor. The only component that needs to be connected is the male end of the JST Connector. Solder the male JST connector socket onto the triad sensor on the backside where the I2C connections are (Fig. 8). The orientation of the JST connector is unimportant as long as the wires are kept track of. The wire colors corresponding to the JST orientation shown in Fig. 8 (right) are in Table 2.

### Enclosure modifications

To make the hard case work for the Weed Warden, holes need to be added to mount the sensor cover, provide a connector pass through, and mount the triad sensor. Optionally, the valve on the front of the case can be drilled to an appropriate size to be replaced with a cable gland to connect the system to power and an external device to be activated by the system.

#### Sensor cover holes in hard case

**Fig. 9:**
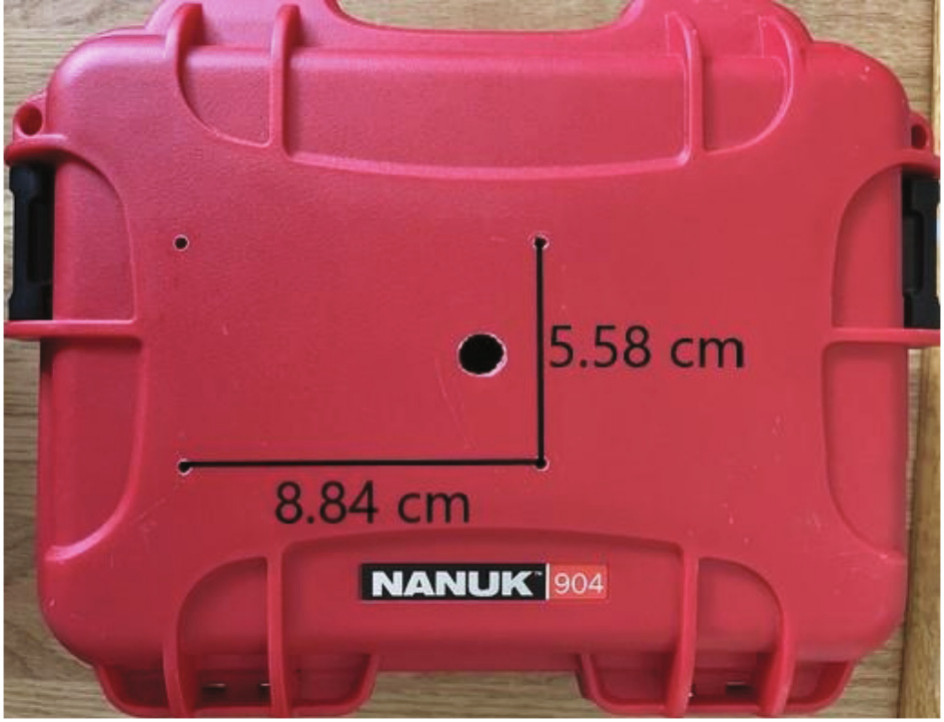
Sensor cover mounting holes with dimensions and wire through hole.

The M3 holes for the sensor cover must be drilled at a spacing of 8.84 cm × 5.58 cm to accommodate the sensor cover (Fig. 9)*.* The sensor cover without screws can be used as a template to draw the hole locations. The locations of the holes on the hard case, other than relative to each other, are not important as long as the holes are all on the flat middle part of the case.

#### Connector and wire hole in hard case

A hole must be drilled in the hard case to allow the JST connector wires through. Drill a 1.3 cm diameter or larger hole in the hard case centered 1.2 cm from the holes for the short edge of the sensor cover and approximately 2.7 cm from the holes for the long edge of the sensor cover (Fig. 9).

#### Triad sensor mounting holes in sensor cover

**Fig. 10:**
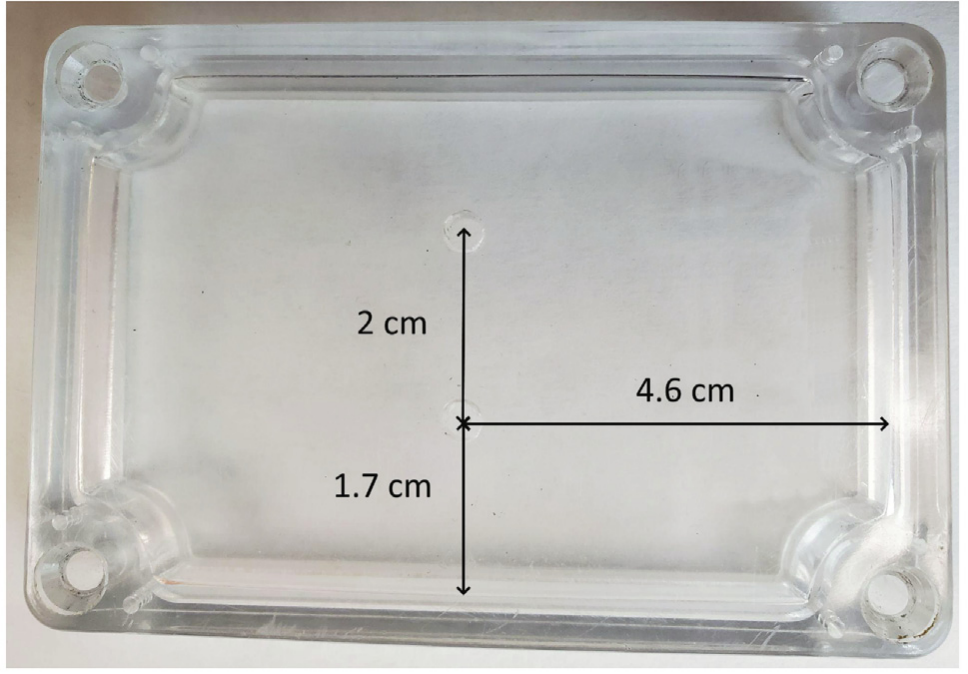
Triad sensor mounting holes with dimensions relative to existing holes on the sensor cover.

The triad sensor must be mounted on top of the hard case underneath the sensor cover. Drill 2 M3 holes in the sensor cover approximately 4.6 cm from the hole centers in one short edge and 1.7 cm from the hole centers in one long edge, while maintaining the proper hole spacing between the holes, 2 cm, for the triad sensor mounting (Fig. 10). Alternatively, these holes could be drilled into the hard case to avoid drilling into the sensor cover, and the Triad sensor would then be screwed into the hard case.

### Final assembly

#### Sensor mounting

**Fig. 11:**
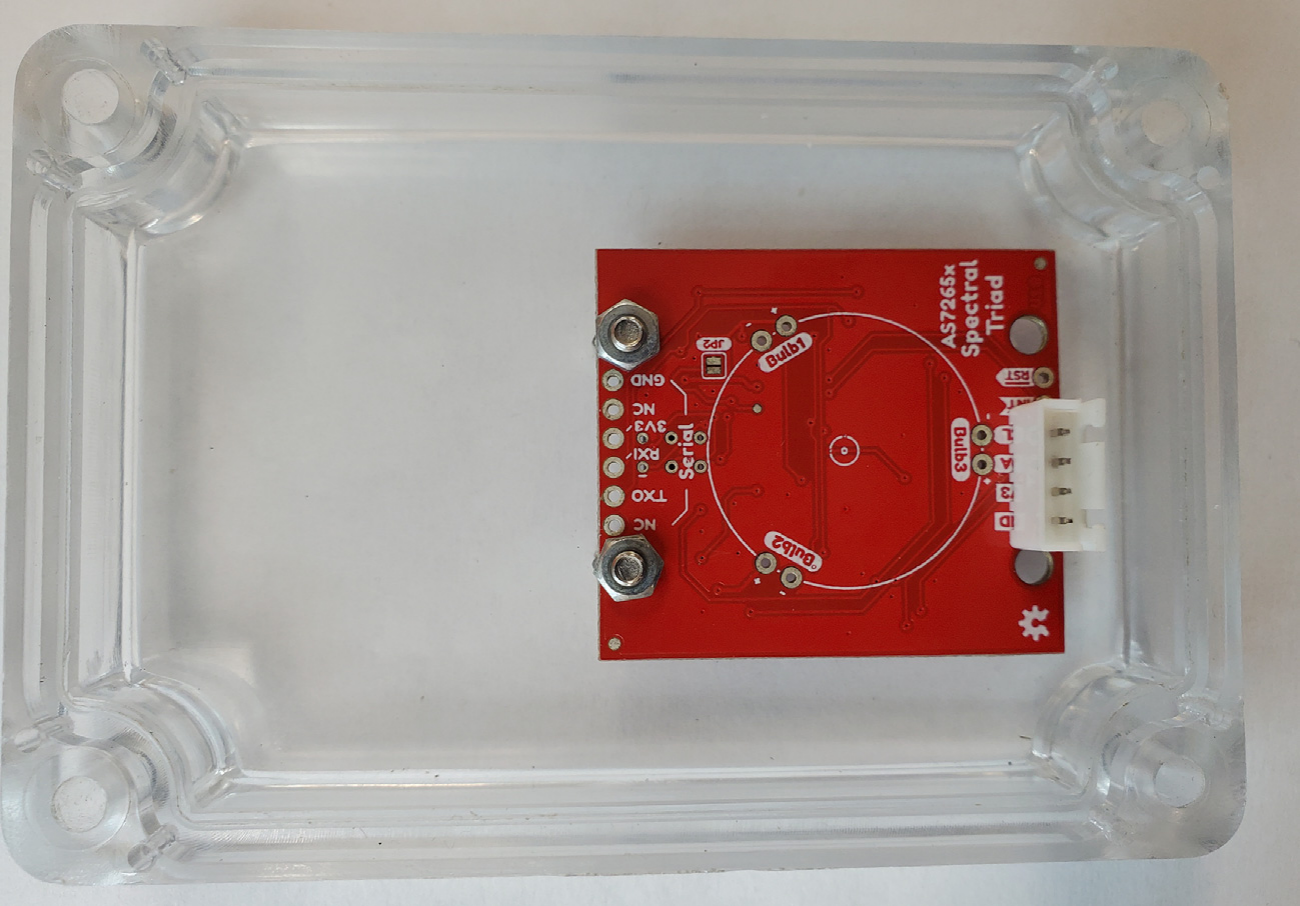
Triad sensor mounted in sensor cover from bottom.

**Fig. 12:**
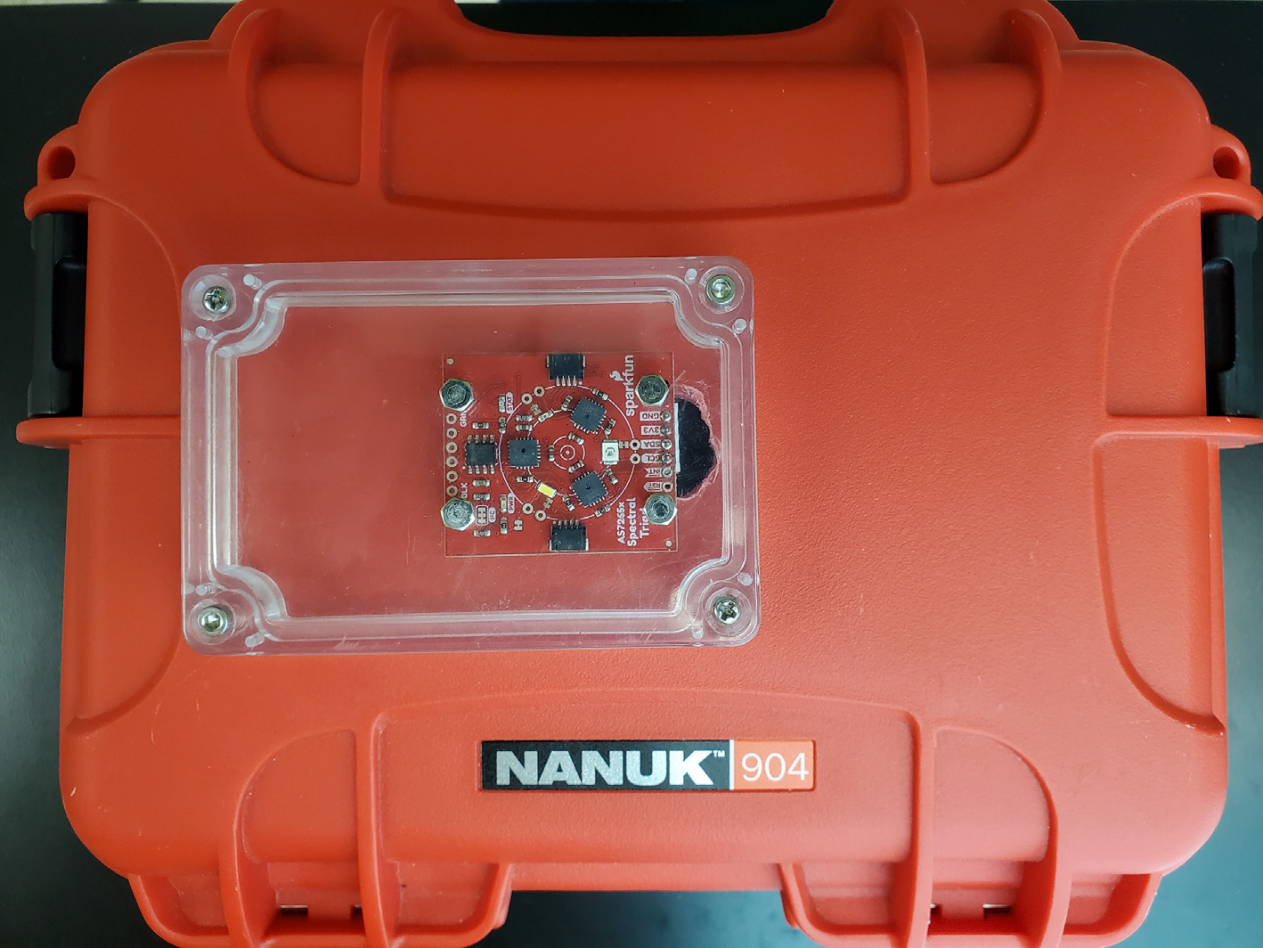
Sensor cover and sensor mounted on hard case. Note two additional optional screws are shown on triad sensor adjacent to the JST connection.

Once the PCBs are connected, the triad sensor and sensor cover must be mounted onto the hard box. Attach two short M3 screws, bolt heads on the outside, with M3 hex nuts to the sensor cover in the holes that were created in step 5.2.3. Then use two additional M3 hex nuts to attach the non-JST side of the triad sensor to the sensor cover (Fig. 11). The first set of nuts is to allow space for the components on the top of the triad sensor. Next, mount the sensor cover to the hard case with four long M3 screws and nuts using the holes drilled in step 5.2.1 (Fig. 12). For waterproofing, use RTV Silicone Adhesive to seal any holes or joints on the system prior to tightening fasteners.

#### JST connection and PCB placement

**Fig. 13:**
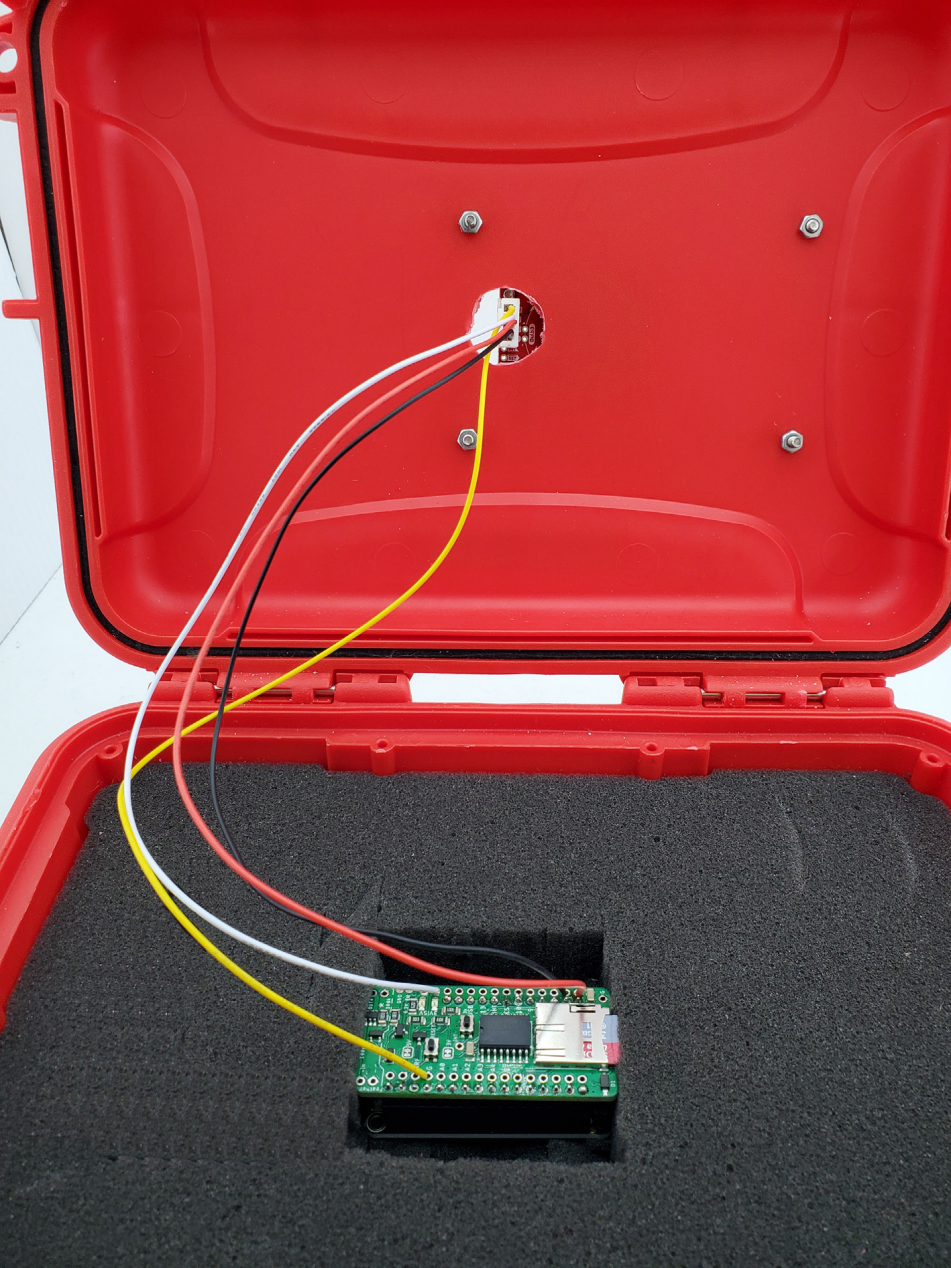
Interior of enclosure with all wires connected. Note cut out for USB cord or battery is not shown.

The final step is to set the stacked Hypnos Board and Feather M0 in the case and plug the JST connector into the Triad Sensor (Fig. 13). Prior to inserting the PCB stack, cut a 5 cm square that is 2 cm deep in the foam of the enclosure plus room for a battery or USB cable. Alternatively, remove all foam and use adhesive PCB offsets to mount the Feather M0 on the bottom of the enclosure.

## Operation instructions

**Programming**: Plug a micro-USB cable into the Feather M0 and plug the other end into a PC that has Arduino IDE installed. Version 1.8.13 was used for our testing. Follow the instructions [19] for setting up Loom (version 2.5.0 is compatible with the Weed Warden code) on the Feather M0 and ensure the configuration file (config.h) that is included in the Zenodo repository is in the same folder as WeedWarden-Main.ino.

Upload the code (WeedWarden-Main.ino) in the ‘Weed_Warden_Code’ folder in the Zenodo Repository to the Feather M0. To upload the code, the correct port must be selected from the tools->port menu. The default index in the code for the Weed Warden is to use ENDVI.

**Fig. 14:**
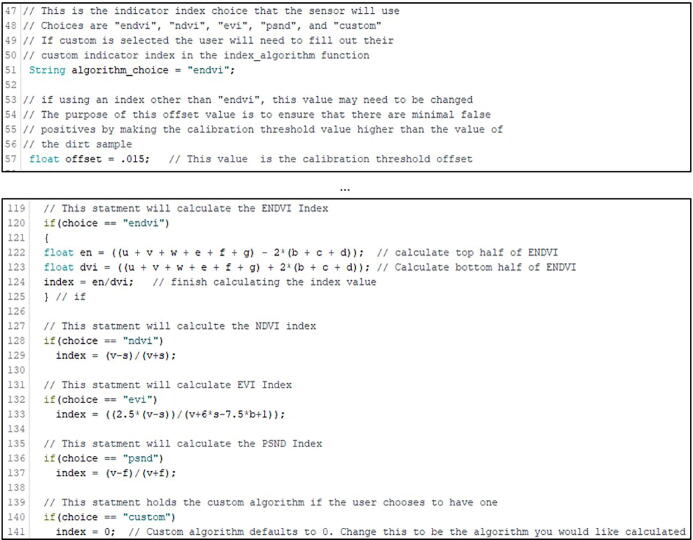
Top: Screenshot of algorithm choice (line 51) and offset value (line 57) in WeedWarden-main.ino. Bottom: Screenshot of index algorithm calculations. Custom algorithm can be input on line 141.

Optional: Prior to uploading, modify the value for the algorithm choice on line 51 or threshold offset on line 57 in WeedWarden-Main.ino (Fig. 14 top). The options for indices are “endvi”, “ndvi”, “evi”, “psnd”, and “custom”. If the user chooses to use the custom index, the user must modify line 141 and set the ‘index’ variable to equal the desired custom algorithm (Fig. 14 bottom). See Fig. 3 or lines 98–115 in WeedWarden-Main.ino for correspondence between variable letters and wavelengths.

**Power and safety:** Power the system either by connecting a 4.2/3.7 V battery to the battery port on the Feather M0 (black 2 pin JST) or a 5 V power supply connected to the MicroUSB port on the Feather M0 [20]. Do not touch the PCB’s electrical connections once it is powered to avoid electrical shock and damage to the components. There is no need to wear any kind of eye protection when working with the triad sensor.

**Deployment considerations:** To determine the appropriate height for deploying the sensing system, the radius of detection, half pixel width, relative to height (Table 3) can be calculated using the field of view angle (20.5°) from the spectral triad sensor datasheet [8]. The system should be deployed such that double the radius of detection is smaller than the expected spacing between plants in order to trigger for each plant, the sensor will clear the tallest expected plants, and the plant width is at least 50% of the radius of detection. Smaller plant width percentages may be successful, but were not evaluated. Heights of 30 and 41 cm were selected for proof of concept testing as they are feasible for tractor or automated rover deployments.

**Table 3:** Radius of detection for triad sensor relative to sensor height, and maximum speed with no overlap.

Height (m)	Radius of Detection (cm)	Maximum speed (cm/s)
0.3	11	11
0.4	15	15
0.6	22	22
0.9	34	34

The maximum speed, which would provide no overlap between pixels, the system should be moved at each height for detection purposes is double the radius of detection divided by the cycle time of two seconds (Table 3). Time for actuation and placement relative to the triad sensor of externally triggered devices (like sprayer or tillage) are important other considerations for maximum speed to move the system.

**Fig. 15:**
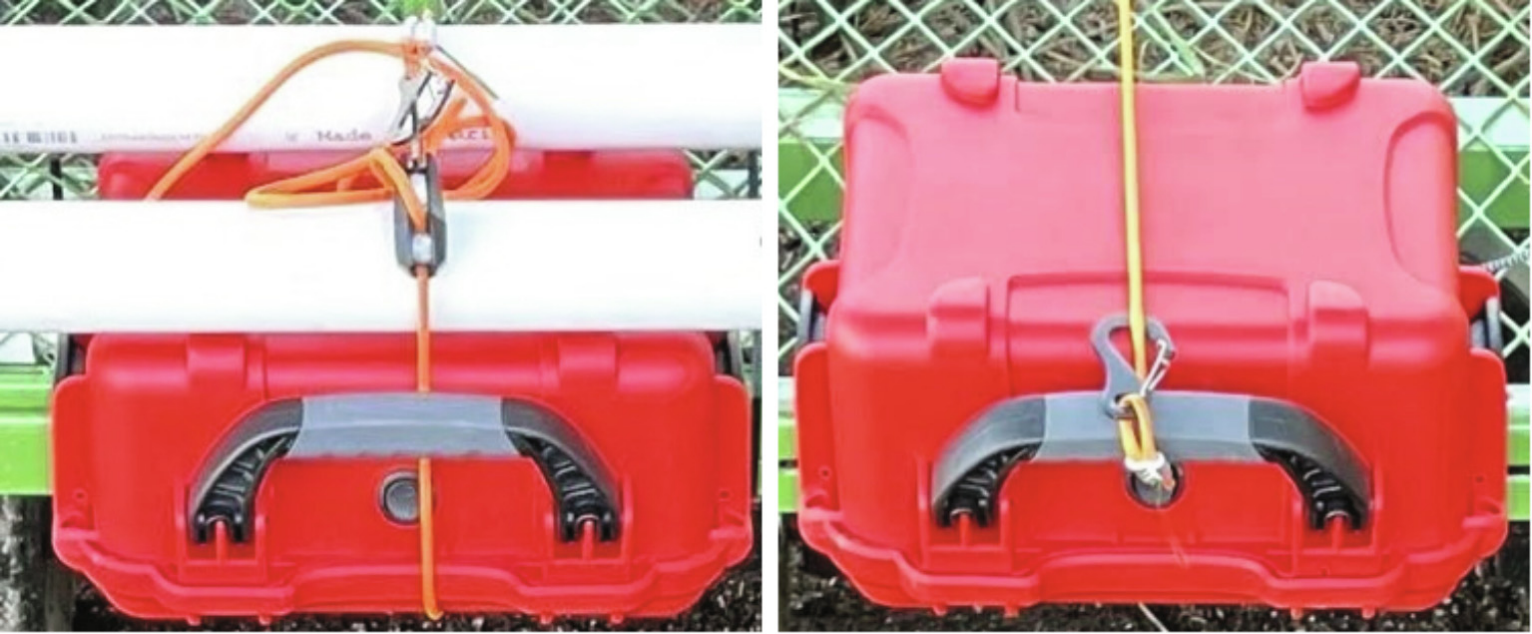
Case mounted on to PVC frame via rope for proof of concept at 41 cm height (left) and 30 cm height (right).

**Mechanical setup:** For the proof of concept testing, the enclosure was mounted to a PVC frame on a cart with a rope (Fig. 15). For future testing and deployment, we recommend using waterproofed screw or bolt attachments through the case mounted to components specific to the vehicle being deployed from.

**Calibration:** Position the sensor at the desired height over a patch of soil that does not have any vegetation present and power on the system. Keep the system stationary and wait for about 30 s for the sensor to calibrate itself. The calibration will take readings of the soil sample, analyze the spectrum for the user-selected charistic index (e.g. ENDVI, NDVI, EVI, etc), and generate a value that will determine where to set the calibration threshold for the subsequently analyzed index values during use to determine the presence of vegetation. This calibration threshold value has an additional offset value added to it to prevent false positives. See section 6.2 for modifying the offset value. Higher offset values will demand a greater detection value for the index relative to soil for plants to be registered. Calibration status can be viewed in the serial monitor. Optionally, a buzzer can be connected between the 5 V rail and ground for an audible cue. The system should be restarted to repeat the calibration process anytime there is a large difference in soil or lighting conditions.

**Operation:** Once the sensor is calibrated, it is ready to detect vegetation. The system automatically loops through sensing, calculating the index, comparing to the threshold, and powering on or off the 12 V relay every 2 s (Fig. 3).

## Validation and characterization

To validate the Weed Warden’s effectiveness the system must be able to:

● Detect vegetation at 41 cm and 30 cm heights.
● Detect all live vegetation samples that are larger than 7.6 × 7.6 cm.
● Log all data to a microSD card.
● Be weatherproof

### Testing procedure

**Fig. 16:**
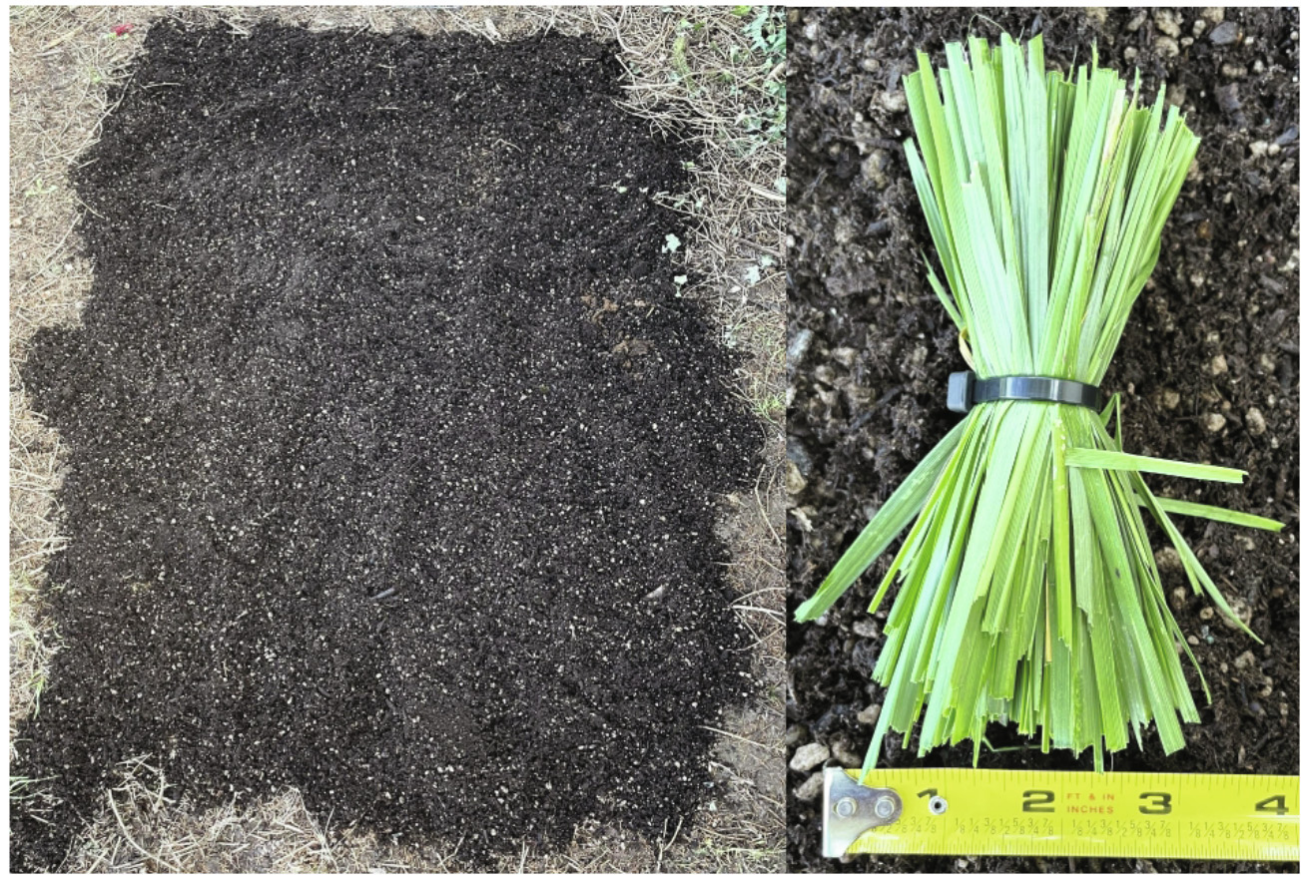
Materials for detection test. Left: A bare patch of soil made of potting soil. Right: Grass sample used for testing.

For these parameters to be tested, a consistent setting was needed. To create a consistent testing environment, a bag of potting soil was used to create a 1.5 × 2.1 m soil surface (Fig. 16 left), and a grass bundle was created to simulate a plant (Fig. 16 right).

**Fig. 17:**
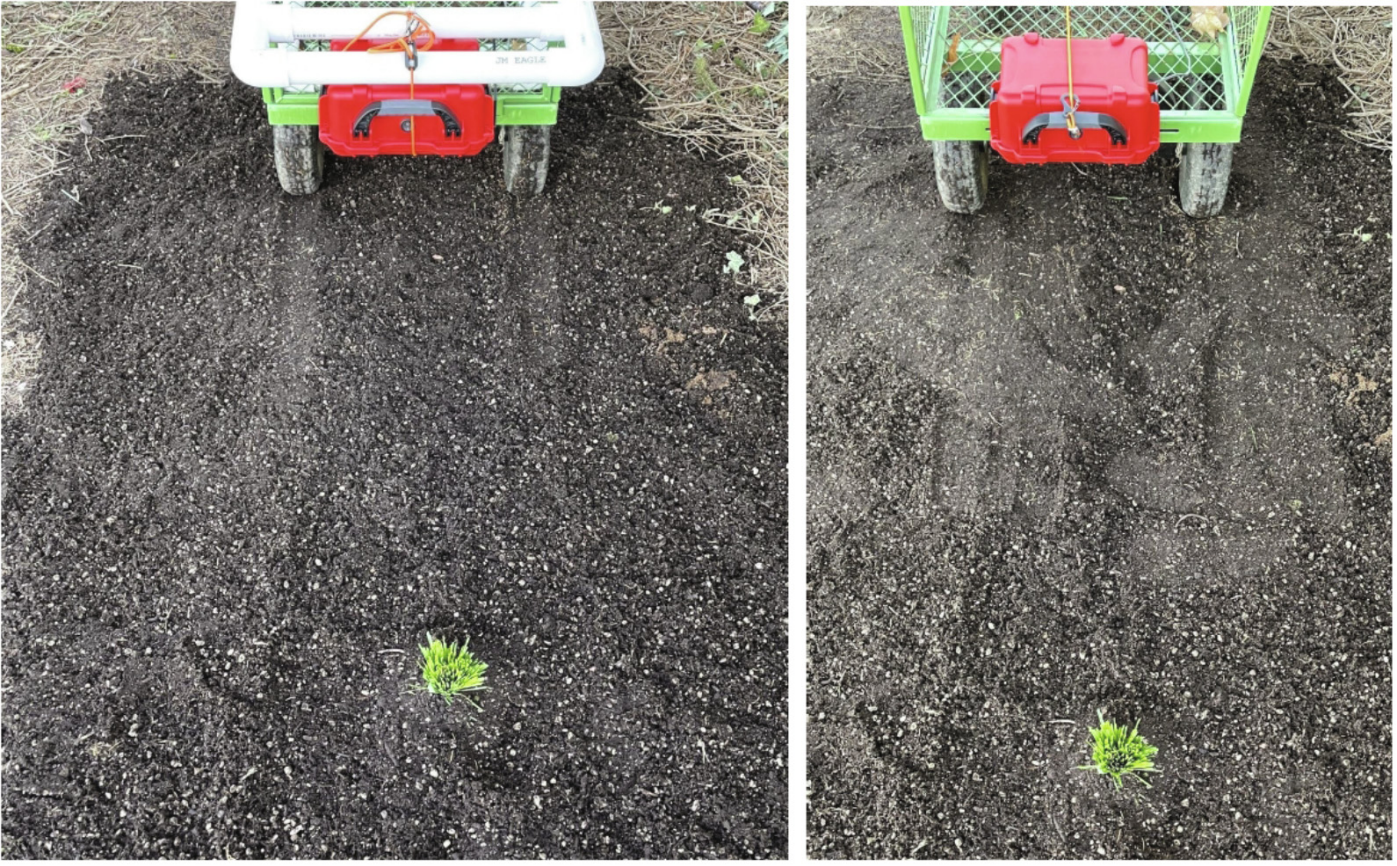
Weed Warden mounted for testing at 41 cm height, left, and 30 cm height, right.

For the final testing setup, the grass sample was ‘planted’ vertically in the soil patch and the Weed Warden was mounted to a cart at 41 cm (Fig. 17 left) and 30 cm (Fig. 17 right) above the soil surface.

Once the physical setup was finished, the Weed Warden was powered on via USB cable from a laptop and the sensor was calibrated over the bare soil patch. A video was also started once calibration was completed so that the video time when the Weed Warden was above the grass could be matched to the data logged to the SD card. After calibration had been completed, the Weed Warden was rolled over the grass sample then back to the soil at a speed of no faster than 10 cm/s. Shaking during testing was minimal. Once three passes over the grass sample had been completed, the Weed Warden was shut off and the video was stopped.

### Results

**Fig. 18:**
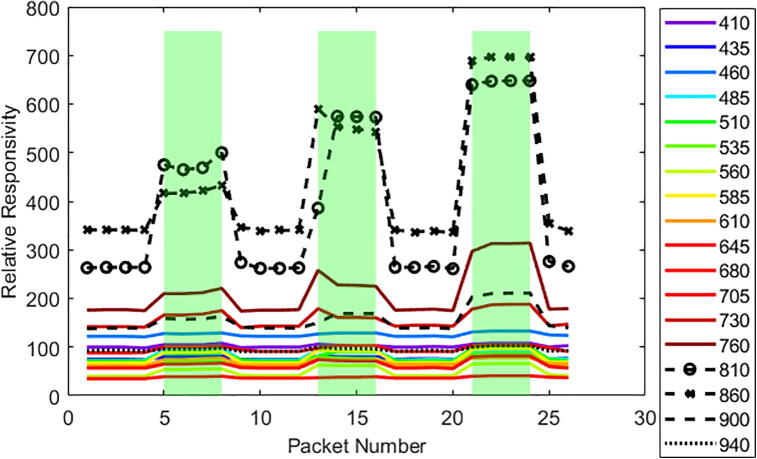
Raw data taken from the triad sensor (lines) and vegetation presence (green shaded area) under the Weed Warden for a 41 cm height test.

**Fig. 19:**
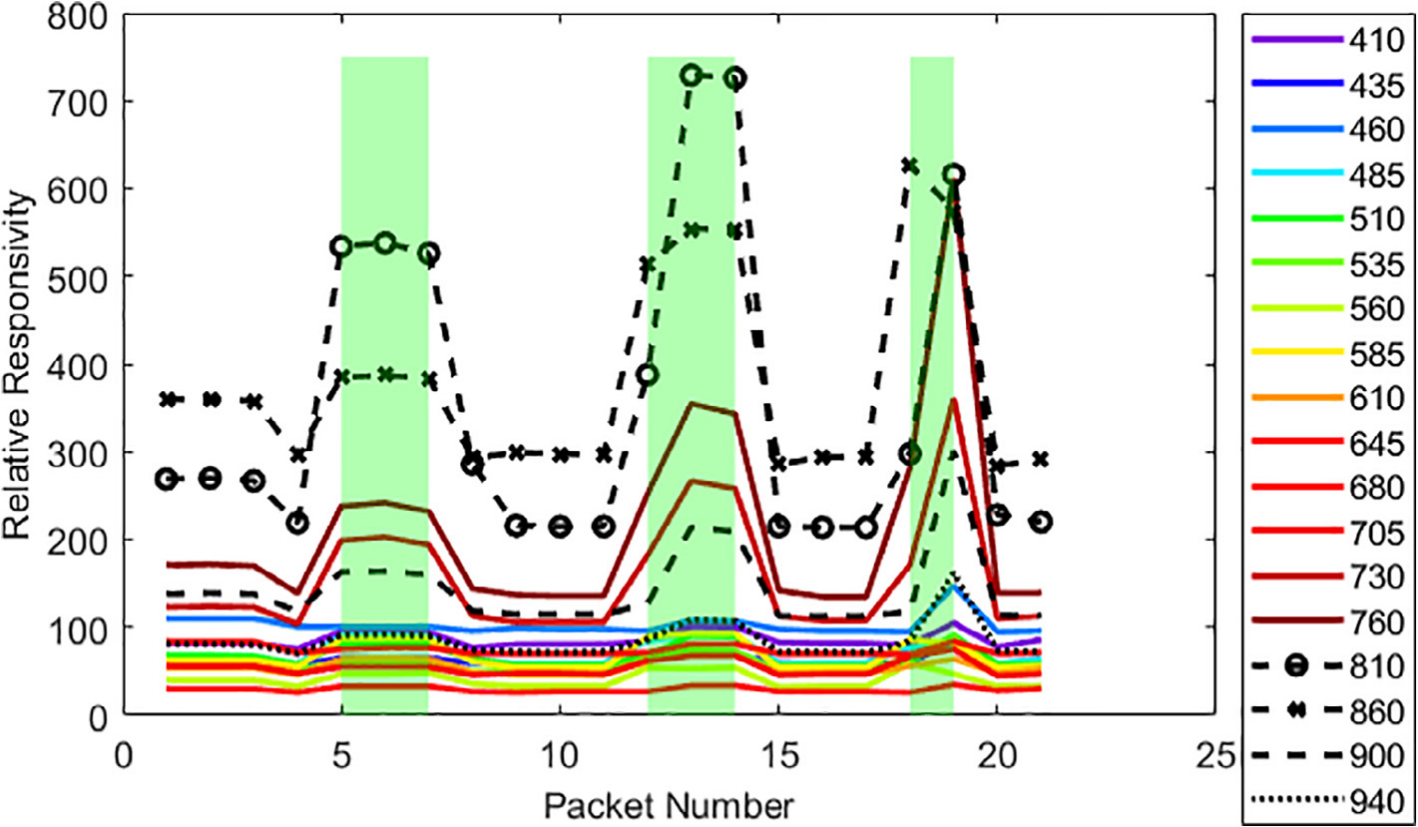
Raw data taken from the triad sensor (lines) and vegetation presence (green shaded area) for the Weed Warden for a 30 cm height test.

Figures 18 and 19 show the raw spectral wavelength data (lines) that was logged to microSD as well as the presence of grass underneath the sensor (green shaded areas) as determined by the video footage taken external to the system. Video footage was matched up to Weed Warden packet number (representing each instance of data collection) by time to show when the grass was present underneath the sensor. The interval between packets is approximately 2 s. The strongest response to both soil and vegetation is in the NIR wavelengths of 810 and 860 for both the 30 cm (Fig. 18) and 41 cm heights (Fig. 19).

**Fig. 20:**
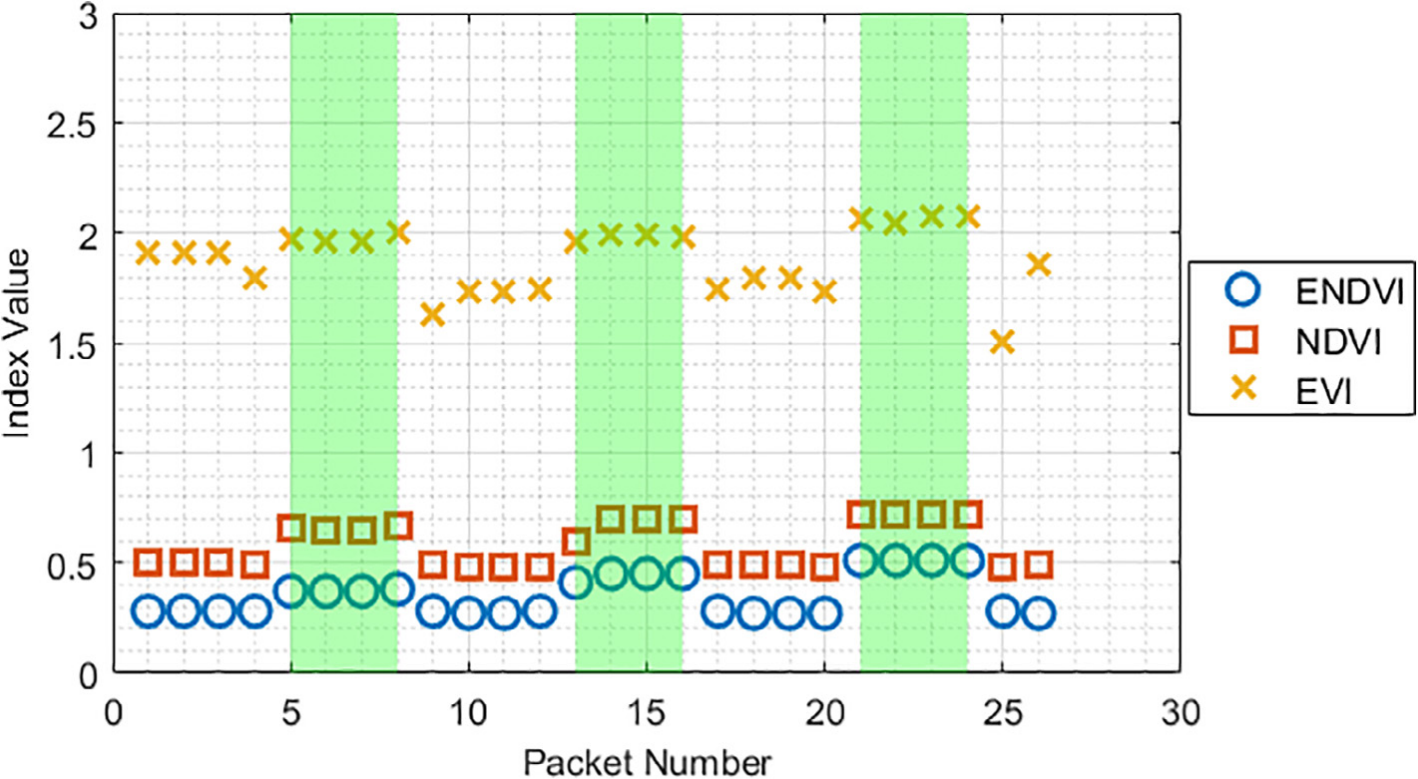
ENDVI, NDVI, and EVI indices plotted alongside vegetation presence (green shaded areas) for a 41 cm height.

**Fig. 21:**
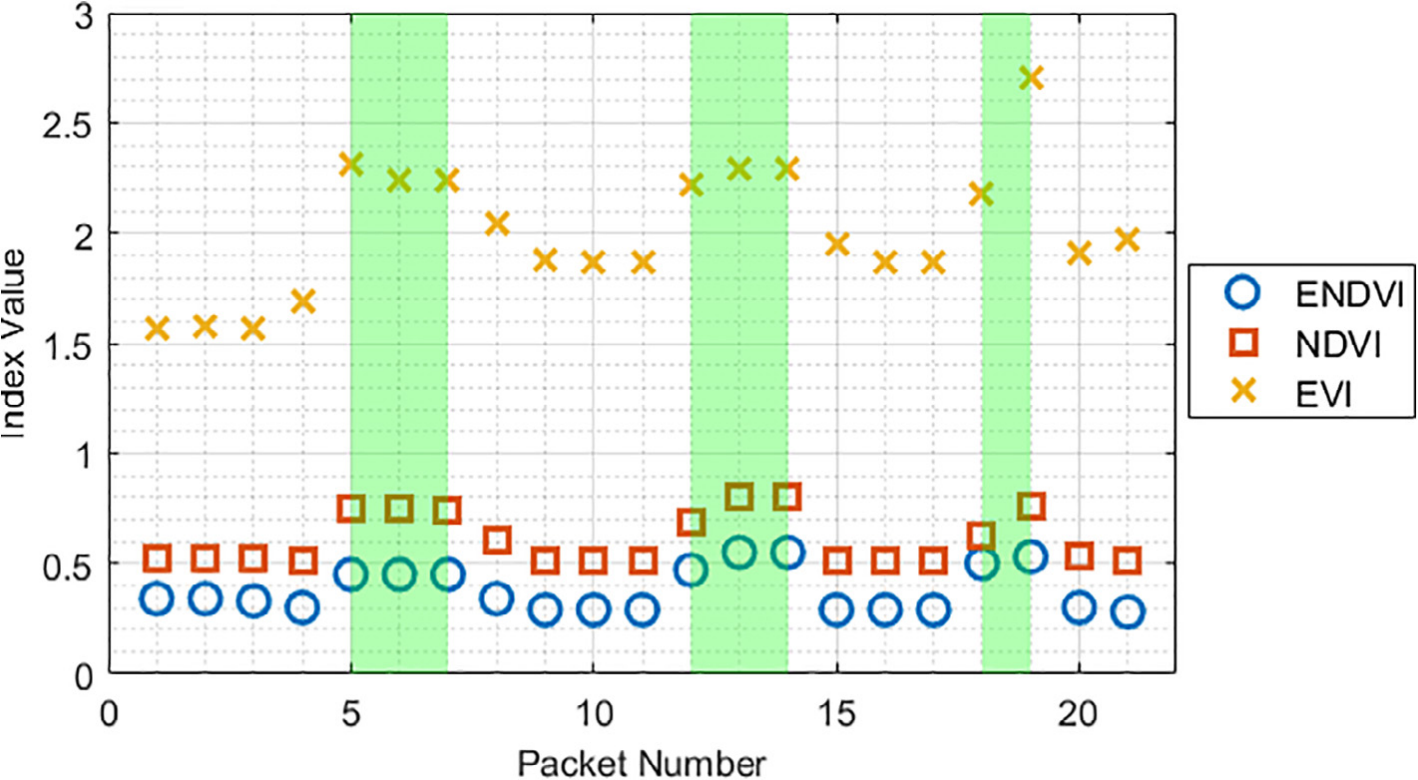
ENDVI, NDVI, and EVI indices plotted alongside vegetation presence (green shaded areas) for a 30 cm height.

During the data collection phase, several algorithms can be used to determine the presence of vegetation under the sensor. The Weed Warden will compute the index of your choice (see Operation Instructions) and compare it to the calibration threshold value to determine the presence of vegetation. Three example indices were calculated for 41 cm and 30 cm height trials with the grass sample (Fig. 20, 21). All three indices showed a noticeable separation between values for soil and vegetation but soil values were more variable for the EVI.

**Fig. 22:**
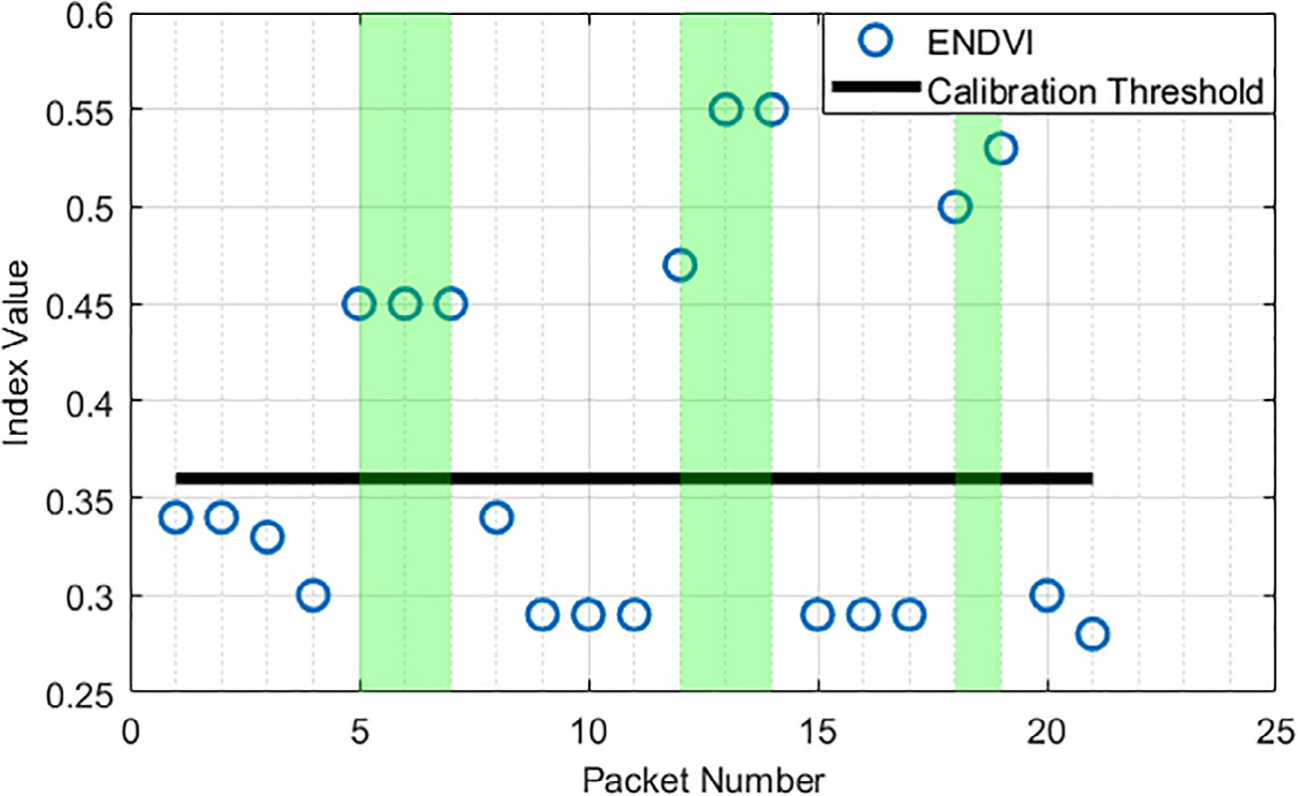
ENDVI index and calibration threshold value for a 41 cm height.

**Fig. 23:**
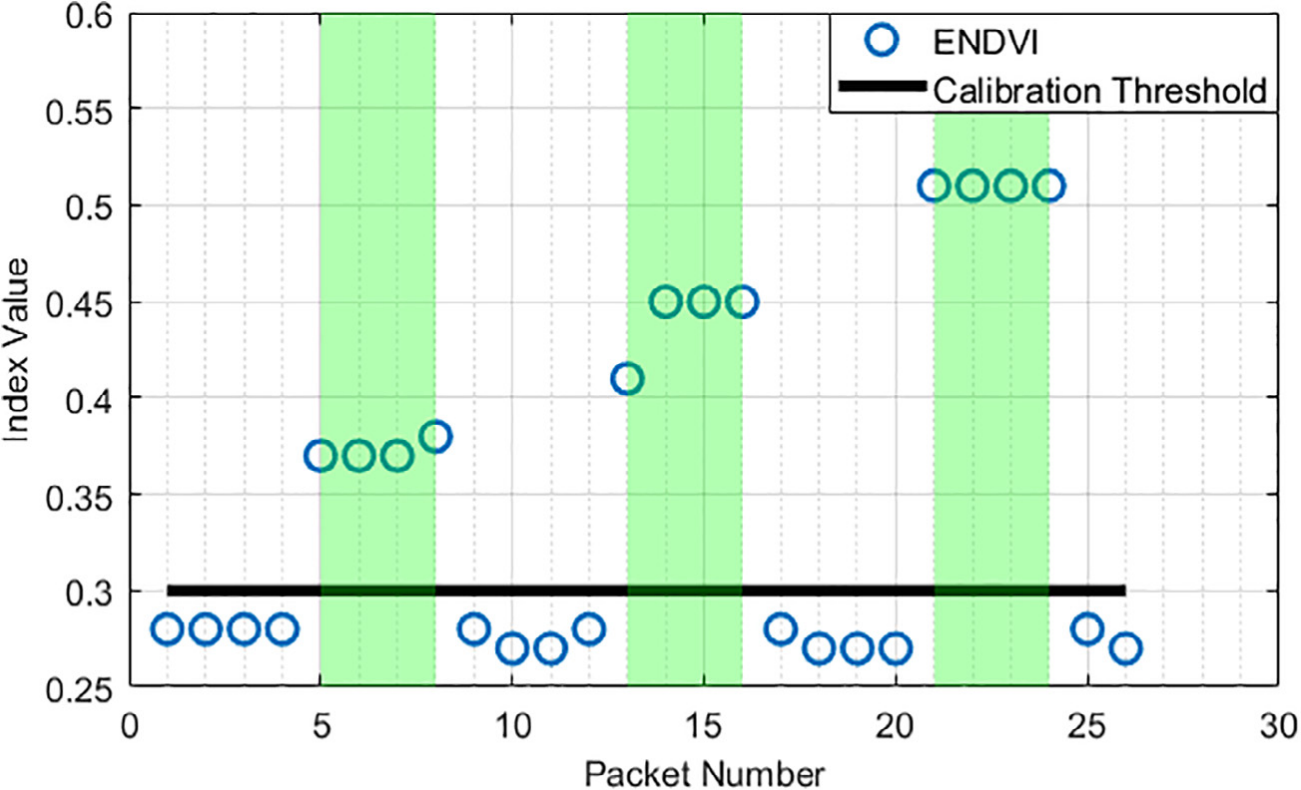
ENDVI index and calibration threshold value for a 30 cm height.

By using the ENDVI and the calibration threshold value, the Weed Warden detected vegetatiofn at every sample the device was above vegetation and at no sample the device was not above vegetation (Fig. 22,23). These charts show ENDVI (blue circles) values with relation to time (represented by packet numbers) and the presence of grass (green area). The chart also shows the calibration threshold value (black line). It is clearly observed that when vegetation is present, the ENDVI crosses the calibration threshold value, which indicates that grass is detected and the 12 V relay is turned on. Note also that the value for the calibration threshold varies by height trial, which is likely due to the ratio of visible vegetation to non-vegetation within the radius of detection changing with the height of the sensor.

By observing the graphs above, the difference in sensing between 41 cm height and 30 cm height can be measured. The data from the 41 cm tests have lower dynamic range across their index values and result in a less obvious detection.

The validation criteria listed at the top of the section were for the system to detect vegetation at a 41 cm and 30 cm height, detect all live vegetation samples that are larger than 7.6 × 7.6 cm in a small testing area, log all data to an SD card, and to be weatherproof. Based on the criteria and the test data above it can be concluded that the Weed Warden is working as intended during controlled testing and is an effective proof of concept. Future plans are to test this device on a land drone or tractor controlling a spray nozzle of neon orange field chalk (the same to mark lines on a football field). We would confirm with manual observation to see how many detected-sprayed areas match against actual vegetation in a test fallow field.

In production settings, a 50′ spray boom can be used to spray several thousand acres every year, with a typical herbicide cost ranging from $10,000 to $20,000. In this scenario, if Weed Wardens reduced herbicide usage by even a fractional margin, it could have significant environmental and financial impact over time. Furthermore, pairing this device with GPS positioning could yield a potentially powerful research tool for studying fallow plant spread to compare weed intervention effectiveness across different strategies (e.g. tillage versus spot herbicide, vs other experimental methods including steam and electricity) in the future.

**Capabilities and limitations**

• Calibration time: 30 s (This can be altered in code)
• Sampling rate: 2 s
• Max Speed: Varies by height (see Table 3): 11 cm/s at 30 cm, 15 cm/s at 41 cm
• 1-pixel cone detection radius: varies by height (see Table 3): 11 cm at 30 cm, 15 cm at 41 cm
• Data collection in 18 bands between 410 and 940 nm
• Real time threshold based triggering according to customizable vegetative index
• General purpose logic signal can send 12 V, 5 V, or 3.3 V to control sprayer, tillage, or other plant removal device

## Declaration of Competing Interest

The authors declare that they have no known competing financial interests or personal relationships that could have appeared to influence the work reported in this paper.

## References

[1] U.S. Fish and Wildlife Service. Impacts of Chemical Methods - Chemical Methods: Management Methods - Managing Invasive Plants. https://www.fws.gov/invasives/stafftrainingmodule/methods/chemical/impacts.html, 2009 (accessed 10.11.2021).

[2] Naïo Technologies. DINO vegetable weeding robot for large-scale vegetable farms. https://www.naio-technologies.com/en/dino/ (accessed 10.11.21).

[3] Trimble. WeedSeeker 2 Spot Spray System - Trimble Agriculture. https://agriculture.trimble.com/product/weedseeker-2-spot-spray-system/ (accessed 10.11.2021).

[4] Laganovska K., Zolotarjovs A., Vázquez M., Mc Donnell K., Liepins J., Ben-Yoav H., Karitans V., Smits K. (2020). Portable low-cost open-source wireless spectrophotometer for fast and reliable measurements. HardwareX.

[5] Chaianantakul N., Wutthi K., Kamput N., Pramanpol N., Janphuang P., Pummara W., Phimon K., Phatthanakun R. (2018). Development of mini-spectrophotometer for determination of plasma glucose. Spectrochim. Acta Part A: Mol. Biomol. Spectrosc..

[6] Lien M.R., Barker R.J., Ye Z., Westphall M.H., Gao R., Singh A., Gilroy S., Townsend P.A. (2019). A low-cost and open-source platform for automated imaging. Plant Methods.

[7] Feyaerts F., van Gool L. (2001). Multi-spectral vision system for weed detection. Patt. Recogn. Lett..

[8] AMS AG. AS7265x Smart 18-Channel VIS to NIR Spectral_ID 3-Sensor Chipset with Electronic Shutter. 2018. https://ams.com/documents/20143/36005/AS7265x_DS000612_1-00.pdf/08051c8a-a7f6-6231-7993-2d3fe0bf38b8 (accessed 10.11.21).

[9] Adafruit. Adafruit Feather M0 Basic Proto. Adafruit Learning System. https://learn.adafruit.com/adafruit-feather-m0-basic-proto/downloads. 2015. (accessed 10.18. 2021).

[10] Nguyen B., Goto B., Selker J., Udell C. (2021). Hypnos board: A low-cost all-in-one solution for environment sensor power management, data storage, and task scheduling. HardwareX.

[11] L. Goertzen et al. OPEnSLab-OSU/Loom: 2.4.1. Zenodo. 2020. doi: 10.5281/ZENODO.4012710. https://zenodo.org/record/4012710/export/csl#.YWR-INrMI_4 (accessed 10.11.2021).

[12] LDP LLC - MAXMAX.COM. ENDVI. https://maxmax.com/maincamerapage/remote-sensing/enhanced-normalized-difference-vegetation-index. 2021 (accessed 10.11.2021).

[13] J. Rouse, R. H. Haas, D. Deering, J. A. Schell, J. Harlan, Monitoring the Vernal Advancement and Retrogradation (Green Wave Effect) of Natural Vegetation. NASA/GSFC Type III Final Report, Greenbelt, Md, 371. (1974). https://core.ac.uk/download/pdf/42887948.pdf (accessed 10.11.2021).

[14] United States Geological Survey. Landsat Missions: Landsat Enhanced Vegetation Index. https://www.usgs.gov/landsat-missions/landsat-enhanced-vegetation-index (accessed 03.01.22).

[15] Blackburn G.A. (1998). Spectral indices for estimating photosynthetic pigment concentrations: a test using senescent tree leaves. Internat. J. Remote Sens..

[16] GS Plastic Optics. Transmission Curves for Plastic Optics. https://www.gsoptics.com/transmission-curves/, (accessed 03.01.2022).

[17] L. Ada, Adafruit Feather M0 Basic Proto, Assembly. https://learn.adafruit.com/adafruit-feather-m0-basic-proto/assembly, 2022 (accessed 02.25.22).

[18] B. Nguyen, B. Goto, Hypnos. https://github.com/OPEnSLab-OSU/OPEnS-Lab-Home/wiki/Hypnos (accessed 05.05.2021).

[19] K. Kang, Loom Quick Start Guide. https://github.com/OPEnSLab-OSU/Loom/wiki/Quick-Start (accessed 05.05.2021).

[20] L. Ada, Adafruit Feather M0 Basic Proto, Power Management. https://learn.adafruit.com/adafruit-feather-m0-basic-proto/power-management, 2022 (accessed 02.25.2022).

